# Opium in India

**Published:** 1895-05-04

**Authors:** 


### Opium in India.

The joys and sorrows of the opium eater have often been described. By some the whole process has been surrounded by a sort of mystery, and by vague stories of unimaginable pleasures, while by others it has been assumed that the use of a drug which can produce sensations so abnormal must be degrading and enslaving.

It has been left to a Royal Commission to tell the simple truth. In most parts of India the natives are apt to suffer from diseases for which if opium is not an antidote, at least it is the most potent remedy they possess. As regards its efficacy in many of the intestinal diseases which are so common, and in the rheumatic affections which so abound in that country, there can be no doubt. The belief also in its power to counteract the evil influence of malaria is widespread, and seems not entirely devoid of scientific foundation. There seems then to exist in India a general consensus in favour of the use of opium as an ordinary domestic remedy in the ailments and diseases common in that most trying climate.

Summing up this side of the question the Report of the Royal Commission says: "It is taken in cases of specific disorders, such as rheumatism, diabetes, chill, and diarrhœa. It is regularly administered to unweaned children. In malarial and damp tracts there is a general faith in its virtue either in warding off or in curing fever. . . . Most of the witnesses with experiences of the rural tracts spoke to the popular belief in the efficacy of the drug in cases of fever or as a preventive against malarial influences." Under these circumstances we can quite understand the Commissioners declining to recommend any further interference with the growth and manufacture of a drug, the use of which as a domestic remedy is so highly esteemed by the people.

There is, however, another side to the question. Plenty of evidence was given as to the extensive use of opium as a mere stimulant or means of creating pleasurable sensations, or an artificial sense of well being, and as to its employment for that purpose leading in a certain proportion of the cases to excess.

There need be no hesitation in confessing that this habit, if carried on to any large extent, is an abuse of the drug. It must not, however, be forgotten that its use for this purpose verges insensibly into its more legitimate use for the relief of pain and the cure of disease, and that it would be exceedingly difficult to prevent its employment for one purpose without vexatiously interfering with its use for the other. Indeed, the line cannot be drawn; the cessation of pain is itself a pleasure, and there is much reason to believe that just as alcohol is often taken here, not merely for the love of it, but for the removal of sensations which, although not amounting to pain, are still productive of discomfort, so opium is commonly taken in India, even when no disease is present, not for the production of positive pleasure but for the sake of that negative gratification which arises from a lessening of those sensations of malaise so common in a climate so depressing as that of India often is.

At any rate, it seems the fact that a very large number of people in India are in the habit of eating opium, just a little every day, that they do it without apparent injury, and even, according to observers on the spot, with benefit; and that in the great mass of instances the habit does not so grow upon them as to lead to excess. In some cases, however, opium is certainly abused, and in these injury results. It seems clear, however, that this is only exceptional, and that in India the excessive use of opium is looked on much in the same light as is the excessive use of alcohol in England. The Commissioners say, "Our conclusions, therefore, are that the use of opium among the people in India, in British provinces, is as a rule a moderate use, and that excess is exceptional, and is condemned by public opinion." The habit of smoking opium would appear to be of much more recent introduction than the old custom of eating or drinking it, and to be much less common. Public opinion also goes against it, and it is considered a more or less disreputable use of the drug. It is also a habit of disreputable people, and it is open to question whether the evils which appear to result from it are not more due to the character of the people who mostly indulge in the vice and the surroundings amid which it is practised than to any specific action of the drug itself when taken in that form.

